# Editorial: Ultrasound in Oncology: Application of Big Data and Artificial Intelligence

**DOI:** 10.3389/fonc.2021.819487

**Published:** 2021-12-22

**Authors:** Yu-Ting Shen, Wen-Wen Yue, Hui-Xiong Xu

**Affiliations:** ^1^ Center of Minimally Invasive Treatment for Tumor, Department of Medical Ultrasound, Shanghai Tenth People’s Hospital, Tongji University, Shanghai, China; ^2^ Ultrasound Research and Education Institute, Clinical Research Center for Interventional Medicine, School of Medicine, Tongji University, Shanghai, China; ^3^ Shanghai Engineering Research Center of Ultrasound Diagnosis and Treatment, National Clinical Research Center for Interventional Medicine, Shanghai, China

**Keywords:** artificial intelligence, ultrasomics, ultrasound, big data, medicine

With the rapid development of science and technology, big data and artificial intelligence (AI) have ushered in a new era for medicine, especially the medical imaging. AI techniques are particularly applicable to imaging-based domains because the pixel values of the images themselves are quantifiable, which is the primary source of data for training and validating algorithms. Big data-based AI has attracted extensive attention for its superior performance and repeatability in medical image recognition. The application of AI technology can provide new clinical perspectives in complex medical imaging to improve diagnostic and surveillance accuracy (1, 2).

As a flexible imaging method, ultrasound (US), with its unique advantages including radiation-free, real-time imaging and ease of use, is expanding globally into various clinical areas as a first-line imaging modality. Ultrasomics is a field of image analysis with a bright future by extracting high-throughput quantitative data information from images and objectively characterizing and interpreting them for clinical analysis and diagnosis (3, 4). AI algorithm-based high-throughput data analysis of ultrasomics can reduce the high operator-dependent inherent nature of US technology, which clearly facilitates precision medicine with clinical decision systems support (Yin et al.; Kuang et al.). In our previous work (5), we summarized the relevant applications of US based on AI technology in different organs in recent years. The related studies also confirmed that this technology undoubtedly has a broad prospects in the field of US. For example, with a prospective and multicenter study design, we (6) developed an assembled convolutional neural network model for identifying molecular subtypes of breast cancer that could contribute to the clinical management of patients with breast disease. And the adoption of tele-US could improve the quality of breast US examinations for inexperienced US doctors (7). In addition, the machine learning-based US visual method (8) achieved an area under the curve (AUC) of 0.9 for the diagnosis of thyroid nodules, effectively reducing unnecessary fine needle aspiration biopsies in the clinical treatment of thyroid nodules. Significantly, the implementation of telemedicine technologies, including tele-US, has shown great value in protecting the health of patients and physicians while providing clinical care decisions for them during the coronavirus disease 2019 (COVID-19) pandemic (9–11). The advanced technologies such as big data analytics, AI technology, 5G networks and the internet of things undoubtedly provide invaluable opportunities for the development of ultrasomics. The main contributions of this Frontiers Research Topic are as follows:

Nowadays, ultrasonography has been endorsed as the first-line imaging modality for diagnosing thyroid diseases by many guidelines (12). Two of the researches Zhang et al. as well as Liang et al. focused on the application of computer-aided diagnosis (CAD) systems to significantly improve the diagnostic accuracy of thyroid nodules, which reduced the need for unnecessary fine needle punctures. It is worth noting that Hou et al. constructed a deep learning-based ultrasonomic model and achieved an AUC of 0.924 for the diagnosis of benign and malignant nodules in patients with Hashimoto’s thyroiditis. Of particular interest is that the study’s dedicated to develop a radiomic nomogram model to non-invasively and reliably predict extrathyroidal extension in patients with papillary thyroid cancer (Wang et al.). These researches all contribute to the response of clinical decision making for precision US medicine.

In recent years, US has been widely used as a significant supplementary modality for breast cancer screening. With the application of AI-based ultrasomics, the diagnostic efficacy of breast disease evaluation has been greatly improved. Xiao et al. studied and demonstrated that deep learning-based computer-aided diagnostic system could significantly outperform the experienced radiologists in terms of diagnostic specificity, sensitivity, and accuracy for patients with asymptomatic breast disease. Benefit from this, unnecessary biopsies in asymptomatic screening patients can be avoided, reducing the waste of medical resources. Another meaningful study designed an ultrasomics feature-based nomogram to predict axillary lymph node status for breast cancer patients with relative accuracy, which helps in clinical decision making (Luo et al.).

It is vital for us to accurately identify gallbladder polyps preoperatively, of which cholesterol polyps and adenomatous polyps are the two most common types, while only adenomatous polyps are true polyps that potentially tend to develop into gallbladder cancer. Yuan et al‘s ultrasomics analysis of spatial and morphological features extracted from raw US images can effectively improve the preoperative diagnosis of true and false gallbladder polyps and provide a reliable basis for clinical decisions related to gallbladder polyp surgery.

Considering that different subtypes of liver cancer determine different treatment modalities and the poor prognosis of hepatocellular-cholangiocarcinoma subtypes, precise identification of liver disease preoperatively is critical. Peng et al. developed a machine learning-based moderate radiomics model to extract high-throughput US image features and achieve superior differentiation of histopathological subtypes of primary liver cancer. In addition, Li et al. conduced a machine learning-based ultrasomics method for analyzing and processing US images, which achieved preoperative individualized diagnostic performance comparable to that of radiologists for patients with atypical hepatocellular carcinoma (aHCC) and focal nodular hyperplasia (FNH). Based on this, the combination of ultrasomics (Nishida and Kudo) approach with AI is gradually becoming an effective tool for the diagnosis and analysis of liver tumors demonstrating the great potential of AI-based ultrasomics applications for precision medicine (13).

It is well known that image feature extraction is the most vital step of data analysis for ultrasomics. To address this critical issue, Jin et al. investigated automatic segmentation algorithms based on the multiple U-net model for ultrasomics features extraction of ovarian cancer patients, which showed good operational reproducibility and reliability. Another research is worthy of our attention is a U-net based on gastric wall detection network developed by Sui et al. By automatically analyzing the hierarchical structure of the gastric wall in gastric ultrasomics, which enable to accurately identify gastric diseases. The data quality and privacy involved in the application of AI in clinical practice and the transparency of algorithms are two important issues that need to be addressed urgently. With the attention and the resolution of these troubles, in the future, AI-based ultrasomics will be considered as a part of routine US examinations that will benefit both patients and physicians. AI technology, encouraged by the demand for ultrasomics application from computer technologies and clinical practice, AI-based ultrasomics will undoubtedly have a broad future. The wide application of AI-based ultrasomics help us to acquire quantify diagnostic information of diseases as well as improve the accuracy and reproducibility of US diagnosis.

We hope that this Frontiers Research Topic will be an enrichment for US medicine, we give our acknowledgement to all authors for their efforts and commitments, as well as the reviewers who have corrected each of the inadequate contributions.

## Author Contributions

Y-TS drafted the Editorial. All authors conceived, designed and supervised this work and gave final approval to the published version.

## Conflict of Interest

The authors declare that the research was conducted in the absence of any commercial or financial relationships that could be construed as a potential conflict of interest.

## Publisher’s Note

All claims expressed in this article are solely those of the authors and do not necessarily represent those of their affiliated organizations, or those of the publisher, the editors and the reviewers. Any product that may be evaluated in this article, or claim that may be made by its manufacturer, is not guaranteed or endorsed by the publisher.
